# Microstructural and Performance Analysis of (TiAl)_95−x_Cu_5_Ni_x_ Coatings Prepared via Laser Surface Cladding on Ti–6Al–4V Substrates

**DOI:** 10.3390/ma17205036

**Published:** 2024-10-15

**Authors:** Wenchang Jia, Xiaojie Song, Yuming Zhu, Di Jiang, Minglei Liu, Yupeng Ji, Dazhou Zhou, Yi Wang

**Affiliations:** School of Materials Science and Engineering, Shandong University of Science and Technology, Qingdao 266590, China; jiawenchang1998@163.com (W.J.); zhuyuming3717@163.com (Y.Z.); jiangdi1210@163.com (D.J.); lml19990218@163.com (M.L.); 17860727609@163.com (Y.J.); 15763675329@163.com (D.Z.); shiwangyi123@163.com (Y.W.)

**Keywords:** laser cladding, TC4, multi-principal alloy coatings, wear resistance, solid solution

## Abstract

In this study, the surface of (Ti-6Al-4V)TC4 alloy was modified via laser cladding. The elemental composition of the coating was (TiAl)_95−x_Cu_5_Ni_x_, with Ni as the variable (where x = 0, 3, 6, and 9 at.%). Multi-principal alloy coatings were successfully prepared, and their constituent phases, microstructures, and chemical compositions were thoroughly investigated. The hardness and wear resistance of the coatings were analyzed, and the compositions and interfacial characteristics of the different phases were examined via transmission electron microscopy. The analysis revealed that Ni formed a solid solution and a eutectic structure in the Ti(Al, Cu)_2_ phase. These findings provide valuable insights into the coating properties. Moreover, reciprocal dry sliding friction experiments were conducted to investigate the wear mechanism. The results revealed a significant increase in wear resistance owing to the formation of a Ni solid solution and changes in the coating structure. Additionally, tensile tests demonstrated that the tensile strength of the coatings initially increased and then decreased with varying Ni content. By combining these results with various analyses, we determined that the coating exhibited optimal properties at a Ni content of 6 at.%. Overall, this study comprehensively investigated the microstructure and phase transition behavior of these coatings through various analytical techniques. These findings provide valuable guidance for further optimizing both the preparation process and the performance of the coatings. The coatings exhibit excellent wear resistance and could inspire the design of more advanced protective surfaces.

## 1. Introduction

Ti-6Al-4V (TC4) is known for its light weight, high strength-to-weight ratio, and outstanding resistance to corrosion [1]. TC4 is widely used in offshore military, medical, and aerospace sectors, including the manufacturer of engine blades, ships, and drilling rods. TC4 has comparatively low surface hardness and wear resistance. To enhance these properties, surface modification technologies [2,3,4] are commonly used. Although these technologies are efficient and cost-effective for enhancing the properties of TC4, achieving high-quality coatings on TC4 remains challenging.

Laser cladding is frequently utilized to produce coatings owing to its rapid cooling and heating capabilities and minimal heat input. This method produces coatings with low porosity, no cracks, good metallurgical bonding with the substrate, and homogeneous, dense structures. Additionally, the method enables flexible equipment operation and easy automation. Since the advent of high-entropy alloys [5], known for their excellent mechanical properties, wear resistance, and high hardness, laser cladding has emerged as the most commonly used surface modification technology and a popular research topic. Ren et al. [6] applied NbMoTaWTi high-entropy alloy coatings to the TC4 surface to improve the crystal lattice, inhibit grain boundary growth, and create diffuse phases. Consequently, the coating exhibited a 72% increase in hardness and a decrease of 121.9% in the wear rate. Zhang et al. [7] used a mixture of TC4, Ni45, Co-WC, and Y_2_O_3_ hybrid powders to prepare a multi-orbital titanium-based laser melting cladding layer on the TC4 surface. The structural characteristics of TiC, TiB_2_, Ti_2_Ni, and matrix α-Ti composite phases were investigated. The results revealed an increase in the hardness of the coatings. Additionally, the coatings exhibited a lower wear rate of 47.8% and an average friction coefficient of 10.5% compared to TC4 substrate. The dominant wear mechanism was abrasive wear. TiAl metal matrix is often used to modify TC4 surfaces owing to its similar benefits, features like high specific strength, low density, and compatibility with the operating environment. Li et al. [8] reported significant improvements in coating performance by melting TiAl/TiC composite coating on the surface of TC4. Zhu et al. [9] used laser fusion cladding to apply a TiAlCu/NbC composite coating to the TC4 surface, which increased the coatinghardness from 642.3 to 1048.8 HV_0.2_, thereby improving its wear resistance. This enhanced wear resistance was mainly due to the addition of Cu, resulting in increased hardness and enhanced brittleness of the coating. The superior tribological properties of TiAl alloys are crucial for engineering applications. Therefore, this study focuses on cladding a TiAl coating onto the TC4 surface.

However, TiAl alloys are prone to brittleness at room temperature, making them comparable to brittle ceramics. This brittleness limits the potential for further improving the wear resistance of TiAl alloys and results in poor mechanical properties. To address these limitations, Ma et al. [10] enhanced the properties of TiAl alloys by incorporating micro- and nano-sized Ti_2_AlC particles into the TiAl matrix. This reinforcement enhanced the molding properties, compression strength, and cracking resistance of the material. Wang et al. [11] prepared TiAl composites reinforced with dual-scale Ti_2_AlC particles to improve alloy properties, resulting in a 28.3% increase in tensile strength. Moreover, various researchers have proposed that incorporating Cu can enhance the plasticity of TiAl alloys owing to the formation of hard phases from Ti and Cu [12,13], leading to improved coating properties. Similarly, the addition of Ni can also improve the tensile properties of the alloy. Yan et al. [14] successfully prepared Ni-based metal self-lubricating coatings through in situ synthesis of the Ti–Si–C system and NiCrBSi on the Ti6Al4V surface via laser surface cladding. These coatings exhibited a solid lubricating phase of Ti_3_SiC_2_, a ceramic-reinforced phase of TiC, and a silicification-reinforced phase of Ti_5_Si_3_. This approach enhanced the coating hardness, significantly reduced the coefficient of friction (COF), minimized adhesive wear, and resulted in a relatively smooth wear surface. Therefore, TiAl composite coatings can be prepared in a variety of ways to improve the performance of TC4.

In this study, TiAl metal-based coatings were prepared through laser cladding to improve the wear resistance of TC4 via solid solution strengthening [15]. The phase compositions and microstructural phases of (TiAl)_95−x_Cu_5_Ni_x_ (where x = 0, 3, 6, and 9 at.%) coatings with varying Ni content were investigated through various characterization methods and performance tests. The coatings were evaluated for hardness, wear resistance, and mechanical properties, and their microstructures were analyzed to extensively elucidate the mechanism underlying the changes in coating properties. The study concluded with an in-depth exploration of TiAl alloys, revealing the relationship between their microstructure and properties. This research offers a theoretical foundation and technical guidance for optimizing the preparation process and improving the properties of TiAl alloys. Additionally, the findings offer new insights and directions for applying TiAl alloys in aerospace, automotive, and biomedical fields.

## 2. Experimental Procedures

**Figure 1:**
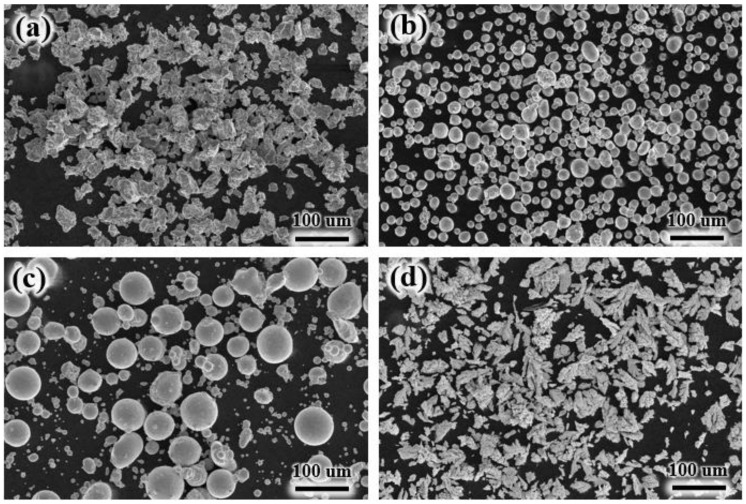
SEM for powders: (**a**) Ti; (**b**) Al; (**c**) Cu; (**d**) Ni.

**Figure 2:**
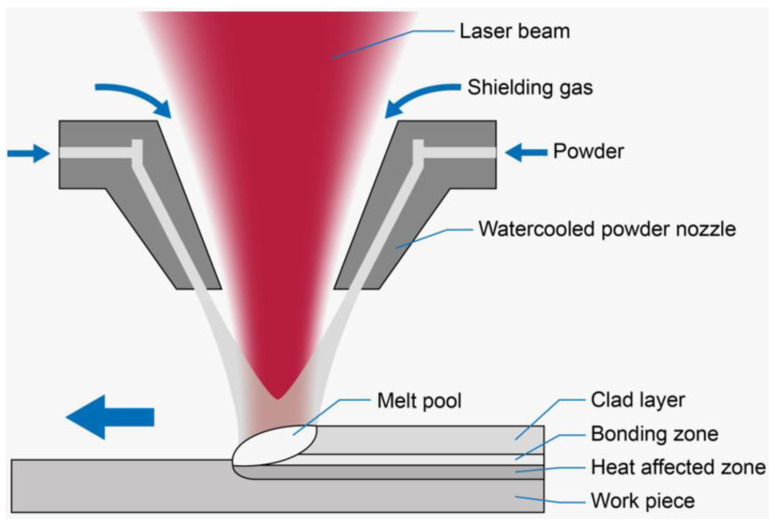
Experimental setup.

TC4 was used as the substrate. Ti, Al, Cu, and Ni metal powders with a purity higher than 99 mass% and a particle size of 40–50 μm were selected and weighed under the equimolar ratio (TiAl)_95−x_Cu_5_Ni_x_ (x = 0, 3, 6, and 9 at.%). Figure 1 shows the morphology of the metal powders, and their specific compositions are presented in Table 1. The metal powders were placed in stainless steel containers with stainless steel balls and alcohol. Following vacuum extraction, the mixture was ground in a ball mill for 12 h. The powder was subjected to drying in a vacuum oven for 48 h. Finally, the alloy powder, measuring 2 mm in thickness and 5 mm in width, was applied to the TC4 surface via the pre-prepped powder method. To ensure a tight bond between the coating and the substrate, the TC4 surface was first polished with 400#, 1000#, and 1500# sandpaper. Subsequently, the TC4 surface was placed in a beaker with alcohol and cleaned using an ultrasonic cleaner to remove surface impurities. Before cladding, the TC4 surface was covered with an oxidized film, which could negatively impact the quality of the laser cladding and the metallurgical bonding between the substrate and the coating. These contaminants were removed using alcohol, as confirmed by similar experiments. The cladding process was carried out using a fiber semiconductor laser with a maximum output power of 2 kW and an operating power of 1500 W. The process parameters included a scanning speed of 5 mm/s, a spot size of 4 mm, and an Ar gas flow rate of 5 L/min. A schematic diagram of the laser cladding is shown in Figure 2. This laser melting equipment, designed for high-speed laser melting with rapid heating and cooling and minimal heat input, is well-suited for melting TiAl. The resulting melted layer has low porosity and forms a metallurgical bond with the substrate. The coating is uniform and dense. The equipment allows for both powder feeding and spreading, with the substrate placed at the lowest position. The experiment is conducted in an argon environment, utilizing a high-energy laser beam for the melting process.

The samples were carved up into 10 mm × 10 mm × 4 mm blocks using a wire cutter and then polished to metallurgical standards. The treated samples were corroded with Kroll solution (HF:HNO_3_:H_2_O = 1:2:50). The phase compositions of the coating samples were analyzed via X-ray diffraction (XRD, D/MAX-2500PC-type, Rigaku, Japan), with a Cu Kα target (λ = 0.15405 nm) as the radiation source. The coating samples were scanned at a rate of 4°/min over a scanning angle range of 20° to 100°. The phase compositions of the coatings were analyzed with Jade 6 software. The microstructures of the coatings were characterized via field emission high-resolution SEM (FEI Nova NanoSEM 450, Hillsboro, OR, USA), and their composition was examined using X-ray spectroscopy (EDS). Additionally, the microstructure, physical phase, and composition were examined via transmission electron microscopy (TEM, ThermoFisher Scientific Talos F200X G2, Waltham, MA, USA). For TEM analysis, the coating samples were first ground to a thickness of 80–100 μm and then thinned to achieve passivation. Microhardness was evaluated from the coatings surface to the substrate using a Vickers hardness tester (HV, ShiDai, HVS-1000, Beijing, China) with an applied load of 2 N and a loading time of 15 s. Hardness assessments were performed at intervals of 100 μm from the bond line of the coating. The wear behavior of the coatings was evaluated using a reciprocating friction and wear tester (Rect MFT-5000, Laredo, TX, USA). The common grinding balls used in this experimental setup included Al_2_O_3_ and ZrO_2_. Since the coatings contained Al, Al_2_O_3_ balls were excluded to prevent interference from Al in the friction and wear tests. Instead, ZrO_2_ balls, known for their excellent strength and wear resistance, were used. The applied loads were set at 10 or 30 N, and the wear tests were conducted for 30 min. Each sample underwent three separate tests, and the average results were calculated. The two load conditions were chosen for the experiments to evaluate the consistency of wear behavior under different stresses. The COF was recorded, and abrasion morphologies were analyzed via three-dimensional (3D) morphometry (Bruker Contour GT-K1, Berlin, Germany) and SEM. The wear rate formula for the coatings was calculated using the formula W = V/N·d [16], where V stands for the wear volume, N represents the load, and d signifies the reciprocating distance. Tensile tests were performed on the coatings using a universal testing machine (MTS E4.5.305). For each experimental condition, three samples were tested according to the GB/T228-2002 standard, “Methods of Room Temperature Tensile Tests for Metallic Materials”. The average values from these tests were used to determine tensile strength and stretching rate. After testing, the fracture morphologies of the samples were examined via SEM.

## 3. Results and Discussion

### 3.1. Phase Analysis

**Figure 3:**
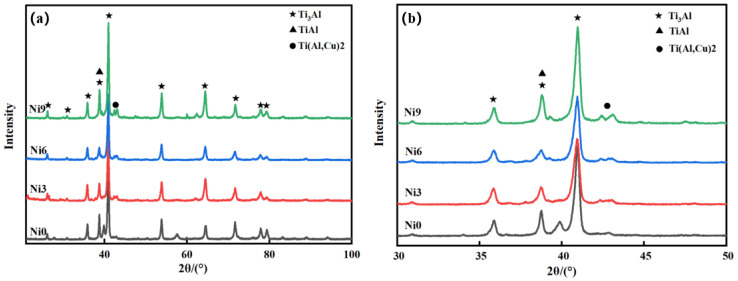
X-ray diffraction: (**a**) full XRD patterns; (**b**) local magnification of XRD patterns.

The compositions of the coatings were examined using XRD, with the results presented in Figure 3. In the absence of Ni, the coatings mainly comprised Ti_3_Al and TiAl phases. Upon the addition of Ni, the Ti(Al, Cu)_2_ phase was formed, as evidenced by the gradually enhanced diffraction peaks. The continuous addition of Ni to the Ti(Al, Cu)_2_ phase led to increased lattice distortion, which improved the properties of the coatings. Moreover, the addition of Ni promoted the formation of the TiAlCu phase, further contributing to these improvements. Consequently, the peaks associated with Ti_3_Al first weakened and then strengthened, indicating that Ti, Al, and Cu combined with increasing Ni content to form the Ti(Al, Cu)_2_ phase. The Ti(Al, Cu)_2_ phase exhibited a hardness of ~550 HV_0.2_, which was enhanced by the solid solution strengthening effect of Ni [17]. This phase typically exhibited a hexagonal Laves phase structure and good interfacial bonding with the substrate, which improved the tensile properties and wear-resistant properties of the substrate material. Additionally, the Ti(Al, Cu)_2_ phase had a modulus of elasticity ranging from ~120 to 150 GPa, indicating its high rigidity and elastic recovery capability. Furthermore, the Ti(Al, Cu)_2_ phase exhibited a high compressive strength ranging from 800 to 1200 MPa. These properties make the Ti(Al, Cu)_2_ phase highly promising for applications in high-temperature structural materials and wear-resistant coatings.

### 3.2. Microstructural Characterization

**Figure 4:**
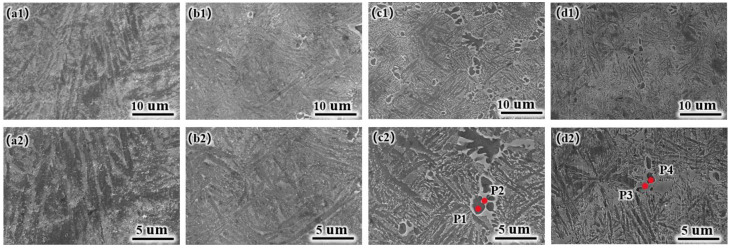
Surface microstructural images of laser-cladded coatings: (**a1**,**a2**) Ni0; (**b1**,**b2**) Ni3; (**c1**,**c2**) Ni6; (**d1**,**d2**) Ni9.

Figure 4 shows the tissue SEM morphology images of the laser-cladded coatings. As the Ni content increases, the slat-like phase gradually transitions to the TiAl phase. The Ti_3_Al phase exhibits more pronounced dark areas, while the Ti(Al, Cu)_2_ phase displays more distinct bright areas. The newly formed phases mainly transition from a slat-like distribution pattern to an interphase distribution between the slat-like structures and the dark areas in the microstructure. Ni is incorporated into the Ti(Al, Cu)_2_ phase to form a solid solution rather than creating a separate phase. To confirm this phenomenon, the different regions of the coatings (Figure 4c2,b2) were analyzed via TEM and EDS. Table 2 shows the elemental composition of the point sweep. The results indicated that the P1 and P4 regions exhibited lower Cu and Ni contents and higher Ti and Al contents than the P2 and P3 regions. The combination of these findings with XRD data indicates that the P1 and P4 regions correspond to the Ti_3_Al phase, while P2 and P3 are identified as Ti(Al, Cu)_2_ phases with Ni in solid solution and some TiAl phases.

**Figure 5:**
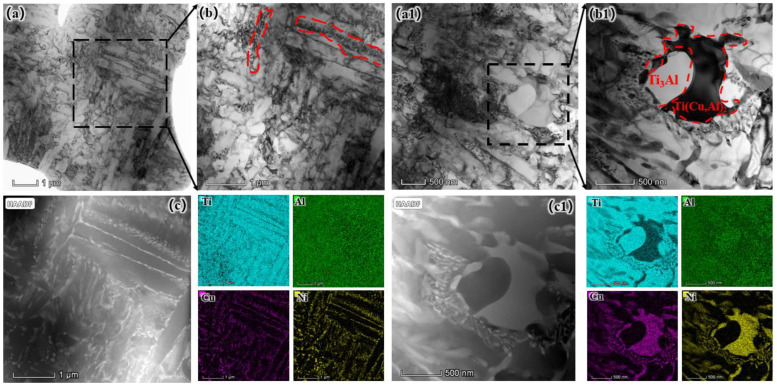
TEM characterization: (**a**) bright-field TEM image; (**b**) local magnification with resilient strips; (**c**) TEM–HAADF images with corresponding TEM–EDS mapping of the region (**b**). (**a1**) bright-field TEM image; (**b1**) local magnification images with interfaces; (**c1**) TEM–HAADF images with corresponding TEM–EDS mapping of the region (**b1**).

To further confirm this hypothesis, Figure 5 presents a bright-field image of the Ni6 samples. At higher magnification, slat-like physical phases are visible, which contribute to the improved toughness of the coatings [18,19,20]. The addition of Ni reduces the grain size of the coatings, since Ni restricts grain growth. Grain refinement typically improves material stretching rate because fine grains can effectively absorb and disperse stresses [21]. However, this improvement is not evident in the SEM images. Further EDS analysis reveals that the slat-like phases consist of Ni, which forms a solid solution in the Ti(Al, Cu)_2_ phase. Figure 5c shows the mapping images of the locally enlarged region shown in Figure 5a. The image display that the area highlighted by the red dashed lines is rich in Cu and Ni, with some Ti and Al present.

To facilitate observation, a larger region of the coatings structure was thoroughly analyzed, as shown in Figure 5b1, which provides a partial enlargement of Figure 5a1. The EDS analysis confirms that the observed coating structure correlates with the structure shown in previous figures. The coating mainly consists of Cu-rich, Ni-rich, Ti-rich, and Al-rich phases. The absence of a distinct Ni phase in the XRD pattern indicates that Ni exists in the coating as a solid solution, dissolved within the Ti (Al, Cu)_2_ phase. The observation of Ni-rich laminar dislocations [22,23] and the presence of Ni-rich zones can be linked to the oscillatory behavior of the laser [24]. The rapid cooling rate induced by the laser results in the formation of elemental-rich zones, resulting in an increased density of dislocations. and laminar dislocations. Although these dislocations are typically considered high-energy lattice defects [25], they contribute to the increased toughness of the coating.

**Figure 6:**
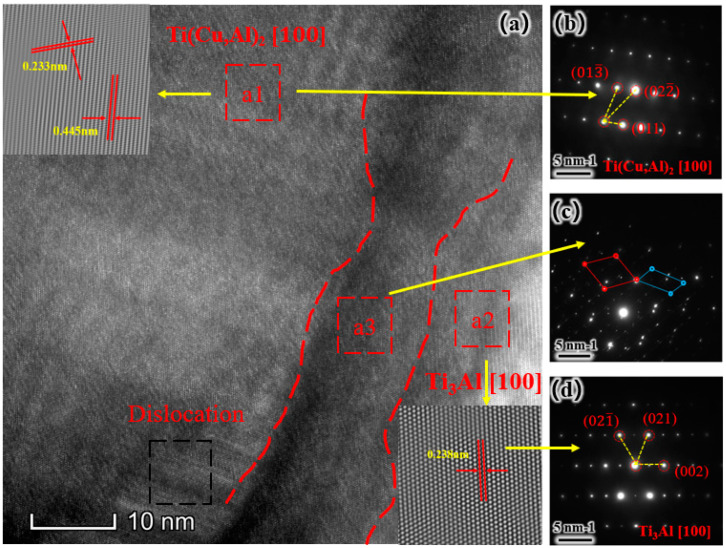
TEM analysis of the Ni6 composite coating: (**a**) High-resolution TEM images of the Ti(Al, Cu)_2_ and Ti_3_Al two-phase interface; (**b**) electron diffraction patterns of a1; (**c**) a3; (**d**) a2.

To further investigate the Cu-rich and Ni-rich phases, Figure 6 presents the Fourier transform map of the region in Figure 5b1. The crystal spacing and electron diffraction maps in Figure 6a reveal crystal spacings of 0.233 and 0.445 nm in Figure 6a1 and 0.238 nm in Figure 6a2. In Figure 6a, dashed lines separate the regions, with the left and right sides corresponding to the Ti(Al, Cu)_2_ and Ti_3_Al phases, respectively. The electron diffraction patterns of the Ti(Al, Cu)_2_ and Ti_3_Al phases are shown in Figure 6b,d. Figure 6c displays the diffraction spots of both phases, indicating a two-phase eutectic region [26]. Figure 6a3 illustrates a distinct interface between the two phases [27]. This interface can hinder dislocation movement and inhibit crack formation, thereby improving the plasticity and toughness of the material. A strong interfacial relationship contributes to the improved performance of the coatings.

### 3.3. Hardness and Wear Analysis

**Figure 7:**
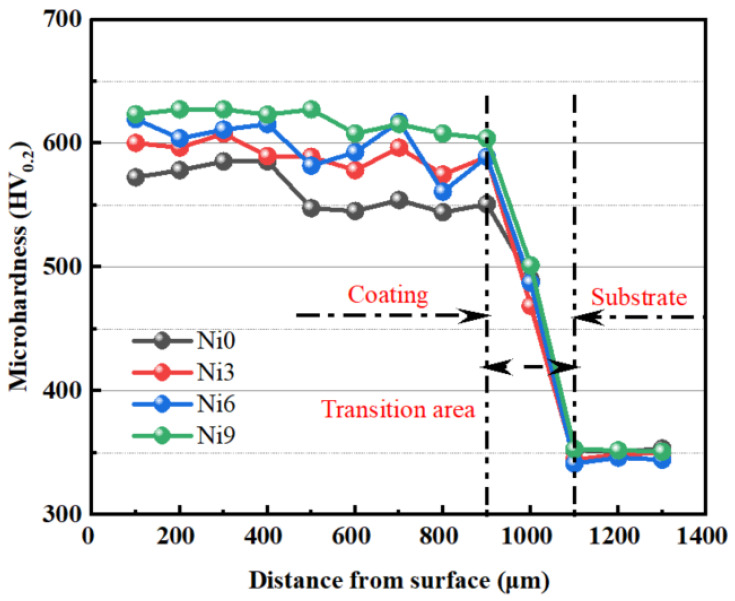
Microhardness of each coating cross-section.

Figure 7 shows the hardness of the laser-cladded coating and illustrates the hardness distribution across the coating, transition zone, and substrate. The transition zone exhibits an intermediate hardness level along the boundary between the coating and the substrate, which aids in buffering stresses. The coating exhibits notably greater hardness compared to the substrate, providing effective protection. Notably, the coating with increased Ni content achieves a maximum hardness of 627.5 HV_0.2_. This increased hardness may be attributed to Ni-induced changes in the lattice structure of the coating, which affects its mechanical properties. Moreover, at a microscopic level, the addition of Ni alters the phase composition and grain structure of the coating. Ni forms a solid solution with Ti(Al, Cu)_2_, resulting in an effect of solid solution strengthening [28]. The introduction of solid solution atoms can increase the lattice stress field [29] and hinder dislocation movement. The addition of Ni promotes the solid solution diffusion into the Ti(Al, Cu)_2_ phase [30], which alters the lattice constant of the crystal. This enhanced solid solution diffusion increases lattice stress, and the presence of the Ni solid solution in the Ti(Al, Cu)_2_ phase can cause changes in the atomic structure at the grain boundaries. These changes may result in the formation of solid solution phases or precipitates, resulting in a grain boundary strengthening effect [31], thereby improving coating hardness.

**Figure 8:**
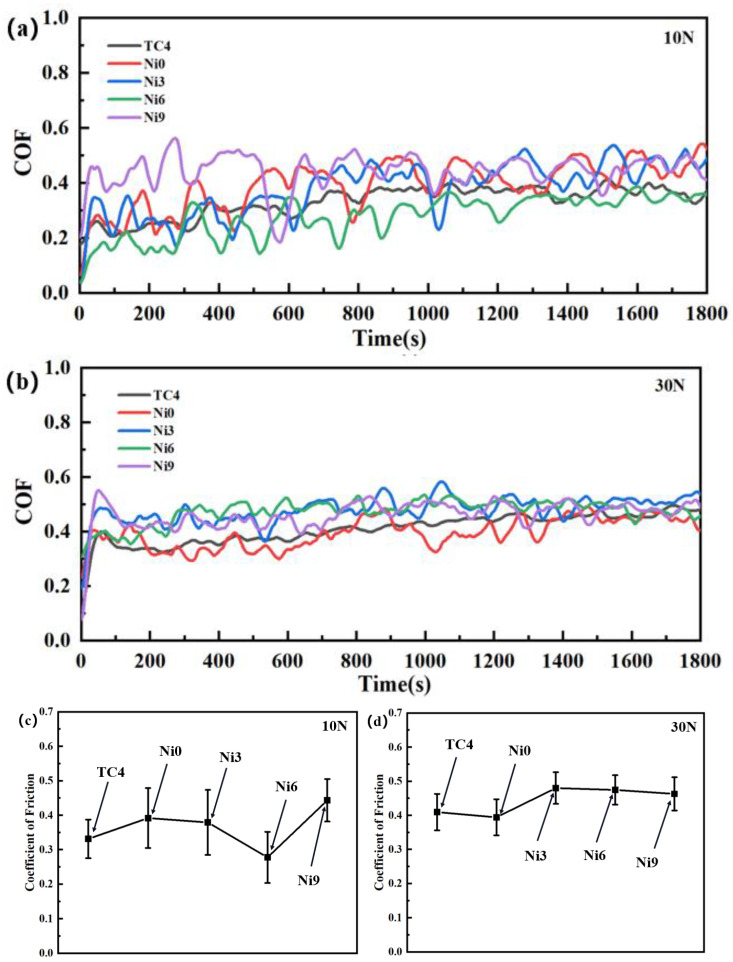
Friction coefficient plots for the specimen and TC4 under varying loads: (**a**) 10 N load; (**b**) 30 N load; (**c**) 10N Average COF; (**d**) 30N Average COF.

Figure 8 shows the reciprocating dry friction experiments of TC4 with coatings under 10 and 30 N loads. The friction coefficient initially decreases before rising with the ongoing addition of Ni. This behavior can be linked to the development of a solid solution with Ni, which slightly reduces the COF. However, as the Ni content continuously increases, the solid solution strengthening effect may reach saturation. Excessive solid solution content can lead to embrittlement of grain boundaries [32], which increases the COF. Additionally, the friction coefficient exhibits less fluctuation under a 30 N load compared to a 10 N load. This reduced fluctuation in friction coefficient may be due to the increased surface roughness of the coating, which stabilizes the coefficient. A larger fluctuation in the friction coefficient indicates that the solid solution in the coating is not susceptible to damage under a 10 N load. During friction, soft phases are prone to damage, which increases the surface roughness of the coating. Additionally, wear debris generated during friction is not easily compacted and removed under a 10 N load, further contributing to the increased fluctuation in the friction coefficient. Under a 30 N load, the friction coefficient fluctuations decrease owing to the compaction of debris, resulting in a more uniform stress distribution across the coating, which facilitates easier compaction and removal of wear debris. Higher loads can cause greater material deformation, which may reduce changes in surface roughness and minimize gaps between the friction surfaces, leading to lower friction fluctuations.

**Figure 9:**
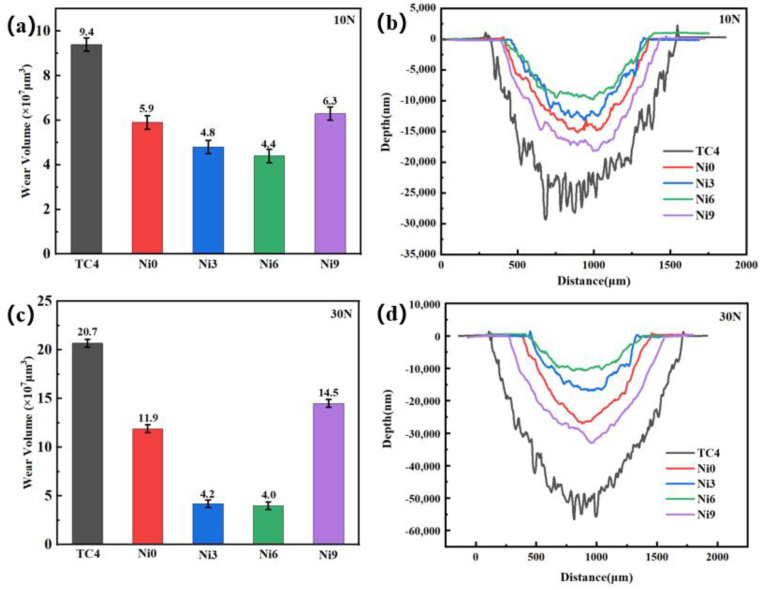
Wear and tear data: (**a**) wear volume of the specimen and TC4 under 10 N load; (**b**) wear profile of each specimen under 10 N load; (**c**) wear volume of the specimen and TC4 under 30 N load; (**d**) wear profile of each specimen under 30 N load.

Figure 9a,c illustrate the wear volume changes under loads of 10 and 30 N. The coatings demonstrate a markedly reduced wear volume in comparison to the substrate. Figure 9b,d present the abrasion profile of the coating. TC4 exhibits the most severe wear. In contrast, the coatings feature a significantly lower wear rate than TC4. Among the coatings, Ni6 displays the lowest wear. The wear volume of the coatings decreases before increasing with higher Ni content. This pattern suggests that the addition of Ni may have optimized the structure of the coating, making it denser or harder, which reduces the wear. However, a further increase in Ni content causes structural instability in the coatings, which impairs their wear resistance. The addition of Ni may inhibit crack propagation at the grain boundaries [33], thereby reducing the wear volume. Nevertheless, higher Ni levels can lead to instability or oxidation at these boundaries, which increases the wear volume.

**Figure 10:**
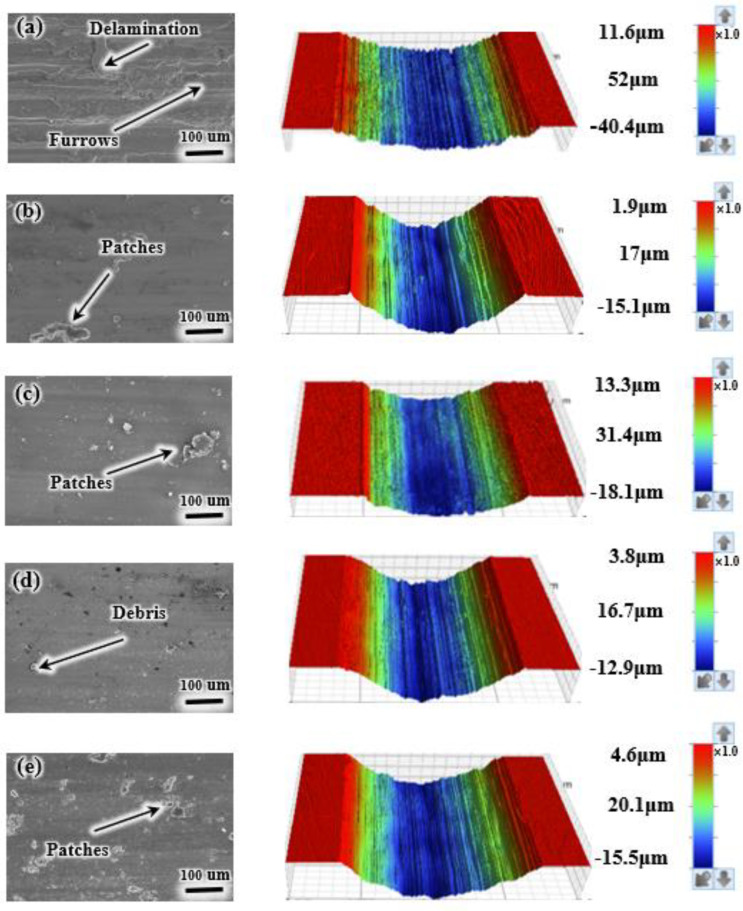
Wear surface morphologies of the specimen and TC4 under a 10 N load. SEM images are shown on the left, and corresponding 3D morphological images are shown on the right: (**a**) TC4; (**b**) Ni0; (**c**) Ni3; (**d**) Ni6; (**e**) Ni9.

**Figure 11:**
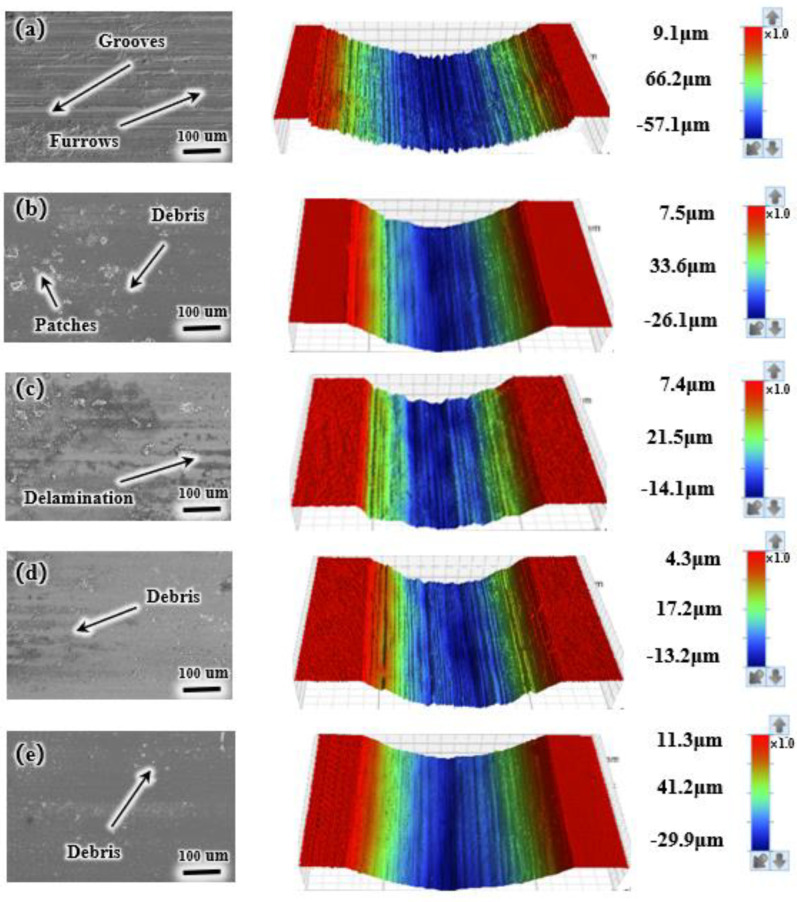
Wear surface morphologies of the specimen and TC4 under a 30 N load. SEM images are shown on the left, and corresponding 3D morphological images are shown on the right: (**a**) TC4; (**b**) Ni0; (**c**) Ni3; (**d**) Ni6; (**e**) Ni9.

To explore the wear mechanism in greater detail, the SEM and 3D morphologies of wear traces under 10 and 30 N loads have been analyzed (Figure 10 and Figure 11). The left image displays an SEM micrograph of the wear scars, while the right image illustrates the corresponding 3D surface morphology, highlighting the wear depth, width, and gully debris. The wear depth and width of the scars correspond to Figure 9b,d. Figure 10a reveals delamination and furrows on the TC4, suggesting mechanical wear and adhesive wear. The prominent mechanical wear mainly contributes to the extensive wear on TC4. In contrast, the coatings exhibit relatively large patches. However, Ni6 features fewer wear marks and minimal plastic deformation. In Ni6, Ni mainly exists in a solid solution within the coating, forming a uniform solid solution with other phases. The solid solution effect enhances the properties of the coating, thereby reducing delamination and stripping on the wear surface. As the Ni content continues to rise, the load-bearing capacity of the coating may be exceeded, leading to structural instability. This instability results in more patchy precipitates on the coating surface, which increases the inhomogeneity and delamination on the wear surface. Additionally, the abrasive scar morphology displays significantly less furrowing and delamination under a 30 N load compared to a 10 N load. This can be attributed to the increased contact pressure at the frictional interface with a higher load, leading to more intimate contact between the coating and the opposing surface during friction. The higher contact pressure may enhance the wear resistance. Under higher loads, the coating undergoes greater deformation and plastic flow, which minimizes inhomogeneities and localized stress concentrations at the frictional interface, thereby reducing furrows and delamination [34]. Moreover, higher loads generate increased frictional heat at the frictional interface, which may increase the localized temperature of the coating surface. This increased temperature may promote plastic deformation and flow at the coating surface, thereby reducing wear surface inhomogeneities and furrow formation.

### 3.4. Tensile Property Analysis

**Figure 12:**
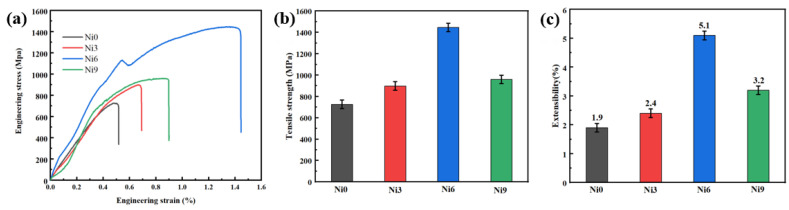
Tensile properties (**a**) stress–strain; (**b**) tensile strength; (**c**) stretching rate.

Figure 12 shows the stress–strain curves for the four coatings, with bar charts illustrating the tensile strength and stretching rates. As the Ni content increases, the maximum stress, tensile strength, and stretching rate of the coatings initially rise and then decrease, reaching an optimal balance at Ni6. A moderate amount of Ni may optimize the grain size and distribution in the coatings, resulting in a dense and uniform microstructure that improves tensile properties. In the coatings, Ni exists as a solid solution and forms a uniform mixture with Ti(Al, Cu)_2_. Similar to the slat-like phase shown in the partial enlargement of Figure 6, this solid solution effect can enhance the plastic deformation ability and tensile strength of the coatings. Excessively high Ni content may exceed the load-bearing capacity of the coatings, leading to structural instability. At low Ni content, increasing Ni can improve the hardness and tensile properties of the coatings. However, excessively high Ni content can reduce the plastic deformation capacity of the coating, leading to reduced tensile properties.

**Figure 13:**
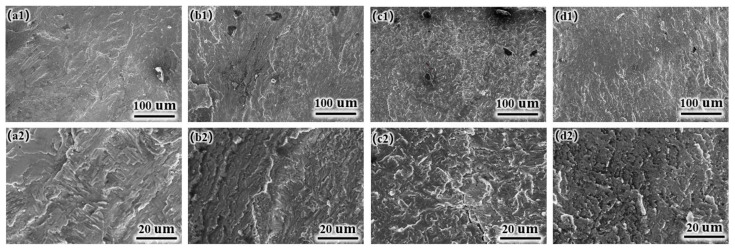
Tensile fracture morphology of the specimens with corresponding local magnification images: (**a1**,**a2**) Ni0; (**b1**,**b2**) Ni3; (**c1**,**c2**) Ni6; (**d1**,**d2**) Ni9.

Figure 13 shows the tensile fracture morphology of the four tensile specimens. Figure 13a1,b1 displays a river-like fracture morphology, characteristic of fractures along the crystal, which are commonly associated with brittle fractures [35]. In contrast, Ni6 exhibits toughness nests, indicative of ductile fracture. However, at Ni9, brittle fracture characteristics are no longer observed.

## 4. Conclusions

Multi-principal element alloy coatings were created on the TC4 surface using laser cladding techniques. The addition of Cu and Ni to TiAl alloy powders improved the wear resistance and tensile properties of the coatings. The following conclusions were reached:

- The coating mainly consists of Ti_3_Al and Ti(Al, Cu)_2_ phases, with Ni fully dissolved in the Ti(Al, Cu)_2_ phase and some TiAl phases, resulting in a multiphase structure that effectively balances strength and toughness. A strong interface is observed between the Ti_3_Al and Ti(Al, Cu)_2_ phases. At a microscopic level, the incorporation of Ni alters the phase composition of the coating. Ni, a solid solution within the Ti(Al, Cu)_2_ phase, induces a solid solution hardening effect, which improves the various properties of the coating.
- Adding Ni to TiAlCu improves the various properties of the coatings through solid solution strengthening. With increasing Ni content, the hardness increases, while wear resistance and mechanical properties initially improve and then decline. Under 10 and 30 N loads, the coatings exhibit significantly lower friction coefficients, smaller width and depth of abrasion marks, and further reduced wear volumes than the substrate, indicating significantly improved wear resistance.
- At the microscopic level, the addition of Ni alters the phase compositions and grain structures of the coatings. Acting as a solid solution in the Ti(Al, Cu)_2_ phase, Ni induces a solid solution hardening effect, further expanding the performance of the coating.
- Tensile tests reveal that with increasing Ni content, the plasticity of the coatings initially rises and subsequently falls. Additionally, the coatings exhibit a ductile fracture, characterized by increased toughness.

## Figures and Tables

**Table 1:** Nominal elemental compositions of Ni0 to Ni9 powders.

	m(Ti)/g	m(Al)/g	m(Cu)/g	m(Ni)/g	Total/g
**Ni0**	17.61	9.93	2.46	0	30
**Ni3**	16.78	9.46	2.42	1.34	30
**Ni6**	15.98	9.00	2.38	2.64	30
**Ni9**	15.19	8.56	2.35	3.90	30

**Table 2:** Composition of elements at each point in Figure 4c2,d2 (mass fraction, %).

Areas	Ti	Al	Cu	Ni
**P1**	64.68	27.70	3.64	3.98
**P2**	54.13	28.09	7.38	10.41
**P3**	52.83	29.60	5.49	12.07
**P4**	65.92	26.64	2.26	5.18

## Data Availability

The original contributions presented in the study are included in the article, further inquiries can be directed to the corresponding author.
